# The prevalence of common skin infections in four districts in Timor-Leste: a cross sectional survey

**DOI:** 10.1186/1471-2334-10-61

**Published:** 2010-03-10

**Authors:** Milena ML dos Santos, Salvador Amaral, Sonia P Harmen, Hayley M Joseph, Jose L Fernandes, Megan L Counahan

**Affiliations:** 1 Communicable Diseases Centre, Ministry of Health, Caicoli, Democratic Republic of Timor Leste; 2 World Health Organization, Caicoli, Democratic Republic of Timor-Leste; 3 School of Public Health, Tropical Medicine and Rehabilitation Sciences, James Cook University, Townsville, Australia

## Abstract

**Background:**

Skin infections are a common public health problem in developing countries; however, they are rarely managed using a population based approach. Recent data on the burden of skin infections in Timor-Leste are limited. Our survey appears to be the only widespread survey conducted in more than 30 years and was designed to determine the baseline prevalence of some common skin infections in Timor-Leste.

**Methods:**

We conducted a cross sectional survey in 14 sites including community health clinics, schools and hospitals within four different geographical regions. Participants were examined for five conditions (scabies, pyoderma, fungal infections, leprosy and yaws) by a multidisciplinary team. Analyses were conducted using EpiInfo version 6.04d.

**Results:**

We examined the skin of 1535 participants aged between four months and 97 years. The majority of participants were male, aged between 11 and 20 years and had at least one condition of interest (56.0%, 56.0%, and 63.1%, respectively). Fungal infections were the most common presentation (39.0%) and males were more commonly affected than females (42.3% vs 34.0%, respectively, pvalue < 0.0001).

Among those people with more than one condition the two most common co-infections were scabies with either pyoderma or a fungal infection (38.0% and 32.0%, respectively). The survey identified 29 previously undiagnosed cases of leprosy and six cases of yaws.

**Conclusions:**

Our findings indicate the need for a comprehensive programme to address these conditions. There are successful disease control programmes in place within the country and it is hoped a healthy skin programme could be integrated into an established disease control programme in order to maximise health benefits and resources.

## Background

Skin infections such as pyoderma and ectoparasitic infections are common in developing countries, especially in tropical regions [1]. Despite their frequent occurrence, they are often not perceived to be a significant health concern, and with the exception of leprosy, these conditions are rarely addressed using population based disease control programmes. This programming gap is often attributed to competing disease priorities and a lack of epidemiological information on the prevalence and/or severity of these conditions, particularly in resource constrained environments.

The factors generally thought to explain the high prevalence and incidence of common skin infections in developing countries are poverty related and include: a low level of hygiene, including difficulties accessing water; climatic factors; and overcrowding living conditions [1]. All these factors are present in Timor-Leste.

Timor-Leste is newly independent country and six years after independence approximately one million people live in the 13 districts, 65 sub districts and 507 villages [2]. The island of Timor is situated approximately 600 kilometres northwest of Australia in the Lesser Sunda Islands at the eastern end of the Indonesian archipelago and has a tropical climate (Latitude: 8° 49' 60 S, Longitude: 125° 45' 0 E). Timor-Leste includes the eastern half of the island, the enclave of Oe-cusse on the northwest portion of the island of Timor, and the islands of Atauro and Jaco.

The country has a young population with an age structure typical of developing countries. Almost 20% the population is under five years of age, slightly more than half are under 15 years and about two-thirds are less than 25 years old [3]. The vast majority of the population is very poor and reside in rural areas with limited access to infrastructure including comprehensive medical services [2].

Health indicators in Timor-Leste are among the lowest in South East Asia and communicable diseases are significant component of the major health problems [3].

Recent data on the prevalence of skin infections in Timor-Leste are limited. We were able to identify only three studies conducted between 1970 and 2005. During September and October 1970 a dermatological survey was conducted in two sub-districts in the districts of Baucau and Manufahi [4]. Almost 3000 people were examined for a variety of conditions. More than a third of participants in Baucau were found to have a skin disease (38.9%, 553/1505 in Baucau and 19.6%, 295/1421 in Manufahi), where the majority of these had a transmissible skin disease (84.2%, 272/295 in Manufahi and 87.5%, 484/553 in Baucau) [4]. The most common transmissible diagnoses were scabies and superficial fungal infections. Almost two thirds of those examined, in one sub-district, had scabies (61.3%, 181/295 in Manufahi and 30.7%, 170/553 in Baucau). The prevalence of fungal infections was also high (24.0%, 71/295 in Baucau and 49.5%, 274/553 in Manufahi). Data on leprosy cases, by district, diagnosed in 1969 were also presented and in 1969 the rate of leprosy per 10,000 population in the four districts where our survey was conducted were as follows: Oe-cusse 72.8; Cova Lima 7.4; Bobonaro 5.5; and Dili 4.4 [4].

Chevalier et al (2000) reported the first confirmed cases of cutaneous leishmaniasis, a parasitic disease spread by the bite of infected sand flies, in East-Timor and, according to the report, all of Indonesia. They diagnosed 46 cases during free medical clinics conducted in November 1999. The cases were clinically compatible and confirmed by direct microscopic examination of lesion specimens stained with May-Grünwald-Giemsa reagent [5]. Since this initial report there have been no subsequent reports of cutaneous leishmaniasis.

Lim (2005) examined the impact of dermatological infections on the Australian peacekeeping forces sent into Timor-Leste in 2000. He found 25% of all the deployed soldier's medical consultations were dermatological related and that bacterial and fungal infections were the most common presentations [6].

In 2006 the Ministry of Health (MoH) reported 48,221 people had visited health facilities with skin problems (skin ulcers, scabies etc) and more than 50% of these were aged under 15 years [7]. In 2007 a skin condition was indicated as the cause of hospitalisation for 2.5% (516/20237) of all hospital admissions and the incidence of scabies infections for all age groups was 2.4 per 10,000 population. For children under one year the rate was 4.5 per 10,000 population [8].

This paper outlines the findings from a survey conducted in September 2007 in Timor-Leste to examine the prevalence of common skin infections.

## Methods

We designed a cross sectional survey, utilising convenience sampling, to determine the baseline prevalence of common skin infections in Timor-Leste. Participants were examined by a multidisciplinary team from the MoH and World Health Organization (WHO) in Timor-Leste together with The School of Public Health, Tropical Medicine and Rehabilitation Sciences (SPHTMRS) from James Cook University in north Queensland, Australia. The screening was conducted over a four week period during September 2007.

Examinations took place in 14 locations in four geographically diverse districts (Oe-cusse, Bobanaro, Cova Lima and on the island of Atauro within the district of Dili) which were selected using convenience sampling. The 14 locations included hospitals, sub-district community health centres (CHC), health posts and primary schools.

All participants provided oral consent prior to being visually examined for five skin conditions; scabies, pyoderma (superficial bacterial infections), fungal infections, leprosy and yaws (also known as framboesia tropica and pian) using a standard protocol. Adult participants and/or guardians of younger children and infants provided consent via a translator. School children gave consent after being informed by their school's head teacher.

The team conducting the examinations applied common case definitions based on MoH existing definitions. Yaws infections included early and latent infections, a clinical presentation in the absence of skin scrapings was used for scabies and fungal infections of any kind were grouped collectively. All unknown conditions were photographed and examined by staff at the SPHTMRS for confirmatory diagnosis.

All newly diagnosed leprosy patients were enrolled in the national leprosy treatment programme and immediately provided with free medication as per WHO treatment protocols. Those diagnosed with other skin infections were provided with treatment or referred to their closest health centre if recruited at a school.

Approval to undertake the study was given by the Timor Leste Ministry of Health, and WHO. James Cook University Research Ethics Committee approved the study protocol (Human Ethics Research Approval H2374).

Data were entered into a purpose designed database and analyses were conducted using EpiInfo version 6.04d. Participants were given a unique identifier, which was used to reconcile the data and remove any duplicates. While individuals were not counted more than once the number of infections, in some instances, exceeds the number of people screened when participants had more than one skin condition.

## Results

**Figure 1:**
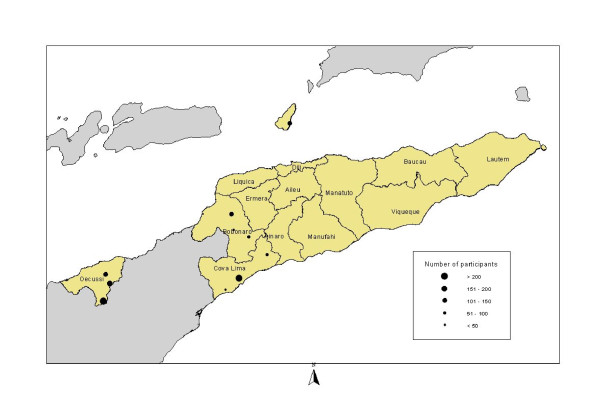
**Number of survey participants by sub district, Timor-Leste, September 2007**.

A total of 1535 participants aged between four months and 97 years were examined. The highest number of participants were recruited in Oe-cusse followed by Bobonaro, Cova Lima and Dili (Atauro) (43.2%, 664/1535; 34.2%, 525/1535; 14.9%, 228/1535; and 7.7%, 118/1535, respectively) (Figure 1). The overall male to female ratio was 1.25:1.0.

The majority of people screened were aged between 11 and 20 years, male and had at least one condition of interest. The most commonly identified condition was a fungal infection and males were more commonly affected than females (male 42.3%, 362/855 and female 34.0%, 231/680, pvalue < 0.0001) (Table 1). The prevalence of fungal infections was high across all age groups although the highest prevalence was found among participants aged between 31 and 50 years where half of those people screened were affected (50.3%, 77/153).

**Table 1:** Infections identified by age group and type, Timor-Leste, September 2007.

Age Group in years	Cases (%)					
	No infection identified	Fungal	Scabies	Pyoderma	Leprosy	Yaws
0 to 5 (n = 100)	18 (18.0)	24 (24.0)	58 (58.0)	30 (30.3)	0 (0.0)	0 (0.0)
6 to 10 (n = 145)	60 (41.3)	44 (30.3)	38 (26.0)	18 (12.3)	1 (0.7)	3 (2.0)
11 to 15 (n = 483)	223 (46.1)	195 (40.4)	71 (14.7)	34 (7.0)	5 (1.0)	3 (1.0)
16 to 20 (n = 486)	254 (52.2)	175 (36.0)	50 (10.3)	22 (4.5)	11 (2.3)	0 (0.0)
21 to 30 (n = 101)	40 (40.0)	49 (48.0)	11 (11.0)	4 (4.0)	4 (4.0)	0 (0.0)
31 to 50 (n = 153)	52 (34.0)	77 (50.3)	27 (18.0)	3 (2.0)	6 (4.0)	0 (0.0)
over 50 (n = 67)	27 (40.3)	29 (43.2)	11 (16.4)	1 (0.9)	2 (3.0)	0 (0.0)

Total (n = 1535)	674 (44.0)	593 (39.0)	266 (17.0)	112 (7.0)	29 (2.0)	6 (0.4)

* Individuals maybe counted more than once if they had more than one condition

Approximately one fifth of all people screened and more than a third of children aged less than 10 years were found to have scabies (17.3%, 266/1535 and 39.1%, 96/245, respectively). The prevalence of scabies decreased with an increase in age. There were 112 pyoderma infections identified, the highest proportion found among the children aged under five years with almost a third of those children affected. The prevalence decreased with age (Table 1).

Approximately 10% all participants were infected with more than one condition and of those 11 individuals were infected with more than two infections (9.4%, 145/1535 and 7.5%, 11/145) (Table 2). When participants were co-infected the two most common co-infections were scabies with either pyoderma or a fungal infection (38.0%, 55/145 and 32.0%, 46/145, respectively).

**Table 2:** Co-infections identified by age group and type, Timor-Leste, September 2007.

	Cases (%)						
Age Group in years	Scabies and pyoderma	Scabies and fungal	Scabies and leprosy	Pyoderma and leprosy	Pyoderma and fungal	Fungal and leprosy	>2 conditions*
0 to 5 (n = 100)	19 (19.0)	5 (5.0)	0 (0.0)	0 (0.0)	4 (4.0)	0 (0.0)	2 (2.0)
6 to 10 (n = 146)	11 (7.5)	6 (4.1)	0 (0.0)	0 (0.0)	2 (1.3)	0 (0.0)	0 (0.0)
11 to 15 (n = 483)	21 (4.3)	15 (3.1)	0 (0.0)	1 (0.2)	6 (1.2)	2 (0.4)	3 (0.6)
16 to 20 (n = 486)	4 (1.0)	9 (2.0)	3 (1.0)	0 (0.0)	7 (1.4)	0 (0.0)	3 (0.6)
21 to 30 (n = 100)	0 (0.0)	2 (2.0)	0 (0.0)	0 (0.0)	3 (3.0)	2 (2.0)	0 (0.0)
31 to 50 (n = 153)	0 (0.0)	8 (5.2)	0 (0.0)	0 (0.0)	0 (0.0)	2 (1.3)	2 (1.3)
over 50 (n = 67)	0 (0.0)	1 (1.5)	0 (0.0)	0 (0.0)	0 (0.0)	1 (1.5)	1 (1.5)

Total (n = 1535)	55 (3.5)	46 (3.0)	3 (0.2)	1 (0.06)	22 (1.4)	7 (0.4)	11 (1.0)

* These co infections have been excluded from other totals e.g., scabies and pyoderma or scabies and fungal infections etc

A higher proportion of females had more than one infection when compared with males (13.0%, 97/768 vs. 7.0%, 62/920, pvalue < 0.0001, respectively).

We identified 29 previously undiagnosed cases of leprosy, the youngest an eight year old female from Oe-cusse. Leprosy was most frequently identified in people aged between 16 and 20 years. Two thirds of the cases were males and the majority of cases were from Oe-cusse (18/29, 62.0% and 19/29, 65.5%, respectively) (Table 1). More than a third of cases with leprosy also had another skin condition (38.0%, 11/29). There were six cases of yaws detected; all male and all were aged between 6 and 15 years (Table 1).

## Discussion

Our survey appears to be the only community based skin survey undertaken in Timor-Leste in more than 30 years. A high proportion of the participants were identified to have at least one of the five skin conditions of interest. These infections (scabies, yaws, leprosy, pyoderma and yaws) cause discomfort and have an acute and possibly long term negative impact on the health of the population.

The findings were consistent with previous studies in developing countries indicating scabies and bacterial skin infections most commonly affect children [5]. Our survey was undertaken at the same time of year as Picoto's 1970 survey and although it was conducted in different districts, the results were similar. Both studies identified skin infections as an important health issue and in particular scabies and fungal infections as the most commonly diagnosed infections.

Despite the high frequency of the diagnoses we believe our results are likely to be an underestimation of the true burden of skin infections and in particular scabies infections in Timor-Leste. In the case of scabies we believe the underestimation is as a result of survey design, transmissibility and environmental factors. Our team most commonly examined only one member of a household as the screening was conducted opportunistically either when a person presented to a clinic or health post for another reason or as part of a school screening. However, when participants were asked about the health of their other family/household members they frequently indicated there were other people in their homes with similar symptoms.

The scabies mite (*Sarcoptes scabei*) is easily transmitted from person-to-person and outbreaks are commonly associated with overcrowded living arrangements and a lack of access to water [9,10]. Both factors are routinely experienced throughout the country. Our survey was conducted toward the end of the "dry" season when water sources in the participating districts are limited further; a situation which could increase the opportunity for household transmission. While it is difficult to determine the extent to which our results underestimate the prevalence of scabies infections we believe, on balance, given the survey design and the environmental conditions more than one case per household could reasonably be expected. Future work including the establishment of a healthy skin programme would need to incorporate examination of household contacts to better estimate the burden of scabies infections.

The high prevalence of people infected with scabies is likely to be an important contributing factor to the correspondingly high proportion of pyoderma observed, particularly among young children. The repeated scratching, caused by a scabies infection, can result in breaks in the skin which in turn facilitates bacterial infections [9].

Group A streptococcus bacterium (*Streptococcus pyogenes*, or GAS) is a bacterium responsible for a variety of conditions ranging from mild superficial skin infections to life-threatening systemic diseases. Worldwide the highest incidence of GAS infections occur in countries with warmer climates, overcrowded housing and where the opportunity to use soap and water is limited [9]. Although our study did not assess whether or not the pyoderma observed was caused by GAS and despite the fact that the prevalence of GAS is not known in Timor-Leste we believe, on balance, that GAS was likely to be a common source of infection. Warranting further investigation for this important public health concern.

The majority of the leprosy cases diagnosed during the survey were from the district of Oe-cusse. Our findings are consistent with both the Picoto's findings in 1970 [4] and the more contemporary findings of the National Leprosy Programme. Timor-Leste has had an active and successful leprosy control programme in place since 2003. The programme primarily focuses on early case detection (via active case finding) and treatment, ensuring all patients have uninterrupted access to multi-drug therapy. As a result of the programme, all districts have reduced rate of leprosy. The overall rate has fallen from 4.7 per 10,000 population in 2003 to 1.7 per 10,000 population in 2007 [11].

As in 1969 the district of Oe-cusse has the highest numbers and rates of leprosy in the country. It has fallen from 54.2 per 10,000 population in 2004 to 13.8 per 10,000 in 2006; however, it still remains unacceptably high [11]. Even with the decrease in the prevalence of leprosy, the proportion of new cases indentified through this study was high (2%, 29/1535) suggesting it is likely that the national prevalence is still underestimated. Further efforts are needed to locate foci with continuing transmission.

While some of the participants we screened were opportunistically examined while presenting at community health centres for other health issues its important to note that most cases of leprosy and all but one case of yaws were discovered through examinations conducted in schools. Thus highlighting the importance of community focussed rather than health institution based control programmes.

## Conclusions

Establishing disease control programmes in a country such as Timor-Leste is challenging. Programmes which require active case finding and subsequent follow-up, such as tuberculosis or leprosy control are time and resource intensive operations.

Yet, despite these limitations there are successful disease control activities being undertaken. Examples include the MoH's integrated programme to eliminate lymphatic filariasis and control of intestinal parasitic infections. It is hoped an expansion of an existing programme to include a healthy skin component can be undertaken. Integrated community based disease control activities allow for shared resources and maximum benefit for the health of the population with a minimal increase in resources. A system of village based health workers are currently being recruited into Timor-Leste's health system. They will be used to support programme implementation across a number of programmes. This public health human resource capacity would be ideal to identify cases of common skin infections to ensure early referral for treatment.

## Competing interests

The authors declare that they have no competing interests.

## Authors' contributions

SA and JF participated in the survey design, logistical arrangements, clinical examinations and data collection. HG participated in the data collection and manuscript preparation. MMS participated in the design, and organization of the survey. MC and SH conceived the survey, participated in its design, collection and analysis of data and manuscript preparation. All authors read and approved the final manuscript.

## Pre-publication history

The pre-publication history for this paper can be accessed here:

http://www.biomedcentral.com/1471-2334/10/61/prepub

## Supplementary Material

Additional file 1 **Data set from Timor Skin Survey, September 2007**. These are the data collected and analysed from the study and presented in the manuscript. Click here for file
